# ITGAL as a Prognostic Biomarker Correlated With Immune Infiltrates in Gastric Cancer

**DOI:** 10.3389/fcell.2022.808212

**Published:** 2022-03-24

**Authors:** Junchang Zhang, Han Wang, Cheng Yuan, Jing Wu, Jiannan Xu, Songyao Chen, Changhua Zhang, Yulong He

**Affiliations:** ^1^ Department of Center for Digestive Disease, The Seventh Affiliated Hospital of Sun Yat-sen University, Shenzhen, China; ^2^ Department of Gastrointestinal Surgery, The First Affiliated Hospital of Sun Yat-sen University, Guangzhou, China

**Keywords:** gastric cancer, integrin alpha L (ITGAL), PD-1, tumor-associated macrophages, prognosis, tumor immune microenvironment

## Abstract

Integrin alpha L (ITGAL) is a member of the integrin family in which the abnormal expression is linked with carcinogenesis and immune regulation. However, the relation between ITGAL and the prognosis of gastric cancer (GC) and tumor-infiltrating lymphocytes (TILs) are not well understood. The differential expressions of ITGAL in human tumors and the clinical prognosis in GC were systematically analyzed *via* multiple databases including Gene Expression Profiling Interaction Analysis (GEPIA), UALCAN, Tumor Immune Estimation Resource (TIMER), and Kaplan–Meier (KM) plotter. TIMER, GEPIA, and TISIDB databases were used to comprehensively investigate the correlation between ITGAL and tumor infiltration immune cells. Also, further results were investigated by immunohistochemistry, qRT-PCR, and Western blot. We found that ITGAL expression in GC samples was considerably increased than in peritumor samples. Sample type, subgroup, cancer stage, lymphatic node stage, and worse survival were strongly related to high ITGAL expression. Moreover, upregulated ITGAL expression was strongly connected with immunomodulators, chemokines, and infiltrating levels of CD8^+^, CD4^+^ T cell, B cell, monocyte, neutrophil, macrophage, T-cell regulatory, NK cell, and myeloid dendritic cell in stomach adenocarcinoma (STAD). Specifically, immunohistochemistry and bioinformatic analysis showed that ITGAL expression was shown to have strong relationships with various immunological marker sets including PD1 (T-cell exhaustion marker). In conclusion, ITGAL is a prognostic biomarker for GC patients. It might regulate tumor immune microenvironment leading to poor prognosis. Furthermore, studies are essential to explore therapeutic targeting ITGAL.

## Introduction

Gastric cancer (GC) is the world’s third leading cause of cancer-related mortality among common fatal tumors (Bray et al., 2018). Despite considerable improvements in diagnosis and therapy about GC, the prognosis still remains challenging (Luebeck et al., 2013). At present, immunotherapy is a prominent subject in the area of cancer treatment and has been an effective treatment in various types of cancer (Larkin et al., 2015; Motzer et al., 2015; Reck et al., 2016), including gastric cancer (Lote et al., 2015; Procaccio et al., 2017). Nevertheless, not all GC patients benefit from immunotherapy, which may be related to the immune microenvironment of tumors (Davidson et al., 2015). Therefore, it is an emergent issue to look for specific immune-related biomarkers with GC and discover new immunotherapy targets.

ITGAL, also known as CD11a, encodes an integrin component of LFA-1, which expressed in immune cells (Silva et al., 2008; Kuwano et al., 2010; Sumagin et al., 2010) and regulated intercellular adhesion and the costimulation signaling of lymphocytes (Ley et al., 2007; Samatov et al., 2013). ITGAL, as a member of the integrin family, which play important roles during angiogenesis and cancer development (Silva et al., 2008; Winograd-Katz et al., 2014; Seguin et al., 2015; Xie et al., 2021), also participates in immune reactions, inflammatory processes, and construction of the tumor microenvironment, thus, contributing to the pathogenesis of diverse tumors, such as renal cancer, colorectal cancer, ovarian cancer, melanoma, prostate adenocarcinoma, and head and neck squamous cell carcinoma (Vendrell et al., 2007; Boguslawska et al., 2016; Song et al., 2019; Zhao et al., 2019; Ji et al., 2020). These researchers suggested that ITGAL might have a significant influence on cancer growth and transformation, and may be a novel target in treating a variety of malignancies. However, the possible mechanisms of ITGAL about tumor development and immune engagement with GC are still unknown.

In this present study, the ITGAL expression and its connection to GC patient prognosis were investigated utilizing diverse databases including the Gene Expression Profiling Interaction Analysis (GEPIA), Oncomine, Kaplan–Meier (KM) plotter, and UALCAN datasets. Furthermore, the Tumor Immune Estimation Resource (TIMER) and immunohistochemistry were performed to investigate the relationship of ITGAL with immune-related cells in the distinct tumor microenvironments. This study uncovered the critical involvement of ITGAL in GC and the possible connection and mechanism by which ITGAL may regulate tumor-infiltrating immune cells.

## Methods

### Tumor immune estimation resource database analysis

The Tumor Immune Estimation Resource (TIMER2.0) is a web-based interactive platform to analyze immune infiltration systematically in various malignancies (https://timer.cistrome.org/) (Li et al., 2020). The TIMER2.0 database applies six advanced algorithms to provide a more rigorous evaluation of tumor-infiltrating lymphocyte (TIL) levels for The Cancer Genome Atlas (TCGA) or tumor-related data. Additionally, the database can also precisely estimate tumor purity. We investigated ITGAL expression in various malignancies and the relationship between the expression of ITGAL and TILs *via* gene modules. Furthermore, the relationship between ITGAL expression with gene markers of TILs, including markers of CD8+/CD4+ T cells, B cells, monocytes, natural killer (NK) cells, dendritic cells (DCs), TAMs, M1macrophages, M2 macrophages, neutrophils, T cells, and related subtypes, has been analyzed *via* correlation modules. Expression dispersion maps were created between a pair of custom genes for GC and the statistical significance of the correlation and estimation of Spearman, by correlation module. The level of gene expression was shown as log2 RSEM.

### Gene expression profiling interaction analysis

The Gene Expression Profiling Interactive Analysis (GEPIA) (Tang et al., 2017) online database (http://gepia.cancer-pku.cn/index.html) is a comprehensive platform that obtained the analysis data from TCGA and The Genotype–Tissue Expression (GTEx) databases. In the present research, we used the GEPIA data source to assess the ITGAL levels in GC samples and healthy samples *via* the “DIY Expression” page.

### UALCAN analysis

The UALCAN website provides an extensive and interactive study of bioinformatics applying RNA-seq and clinical data of 31 malignancies from TCGA (Chandrashekar et al., 2017) (http://ualcan.path.uab.edu/). The database can compare gene expression in tumors to healthy samples, and in different tumor stages or subtypes, as well as other clinicopathological features. This research will, respectively, examine the ITGAL expression lever from major clinical features such as tissue type (healthy/tumor), GC stage (stages 1, 2, 3, and 4), lymph node stage (N0 1, 2, and 3), and cancer subgroup**.**

### Kaplan–Meier Plotter (gastric cancer)

The KM plotter (http://kmplot.com/analysis/) could evaluate the survival prognosis of related genes *via* mapping the survival curve using 1,065 GC samples with an average follow-up of 33 months (Lánczky et al., 2016). The prognostic significance of ITGAL in GC, including overall survival (OS), first progression (FP), and post-progression survival (PPS), was investigated using this database. The hazard ratio (HR) with 95% confidence intervals was also estimated, as well as the log-rank *p*-value. Statistical significance was defined as *p* < 0.05.

### TISIDB

TISIDB (http://cis.hku.hk/TISIDB/index.php) is an online platform that combines various heterogeneous data sources to study tumor and immune system interactions. This database may help researchers understand how tumors and immune cells interact, as well as forecast immunotherapy responses and identify new immunotherapy targets. It would become a valuable resource for cancer immunology research and therapy. In this research, TISIDB was utilized to investigate the association of ITGAL with 28 TILs, 45 immunostimulators, 24 immunoinhibitors, 41 chemokines, and 18 receptors in GC (Ru et al., 2019).

### Real‐time quantitative PCR analysis

According to the instructions, the total RNA of tissue samples was extracted by using MolPure^®^ Cell/Tissue Total RNA Kit (YEASEN CAT#19221ES50). The cDNA of samples was synthesized from 2 µg RNA *via* Evo M‐MLV reverse transcription master mix (Accurate Biology, CAT#AG11706). The qRT-PCR was performed using a SYBR Green Pro Taq HS premixed qPCR kit (Accurate Biology, CAT# AG11701). The ITGAL primer sequence is as follows: forward 5′-CTG​CTT​TGC​CAG​CCT​CTC​TGT-3′ and reverse 5′-GCT​CAC​AGG​TAT​CTG​GCT​ATG​G-3′. GAPDH: forward 5′-CGG​AGT​CAA​CGG​ATT​TGG​TCG​T-3′ and reverse 5′-TCT​CAG​CCT​TGA​CGG​TGC​CA-3′. The 2−ΔΔCT calculation method was used to determine the relative target gene level.

### Western blotting

Protease inhibitors are used to lyse tissue samples in a radioimmunoprecipitation analysis (RIPA) solution. The BCA protein assay kit (KeyGEN BioTECH, cat#KGP903) was used to valuate the protein concentration. Western blot was performed as previously described (Wang et al., 2021). The following antibodies were used: anti-ITGAL (Abclonal, cat#A1644) and anti‐GAPDH (Proteintech, cat#60004‐1‐Ig).

### Immunohistochemistry

This research was conducted on 10 paraffin-embedded GC specimens from the FAHSYSU Department of Pathology. IHC was performed to investigate the expression of ITGAL and PD1, thus, to identify the connection between ITGAL expression and TILs. IHC staining of these specimens were conducted as previously described (Yang et al., 2019). Anti-ITGAL (Abclonal, cat#A1644) and anti-PD1 (Abclonal, cat#A11973) were used for IHC staining. The staining intensity was classified as follows: negative (−), weak (+), moderate (++), and strong (+++).

### Statistical analysis

The KM plots were performed to construct survival curves. For KM plots, GEPIA, and TISIDB, HR and *p*-values were described using log-rank test. Spearman’s correlation coefficient was calculated to analyze the connection of ITGAL expression with immune infiltration levels, immunomodulators, and chemokines. The strength of correlation was judged to be a very weak correlation if <0.2, weak if <0.4, moderate if <0.6, strong if <0.8, and very strong if <1.0. Statistical significance was defined as *p* < 0.05.

## Results

### Aberrant integrin alpha L expression in gastric cancer

**FIGURE 1:**
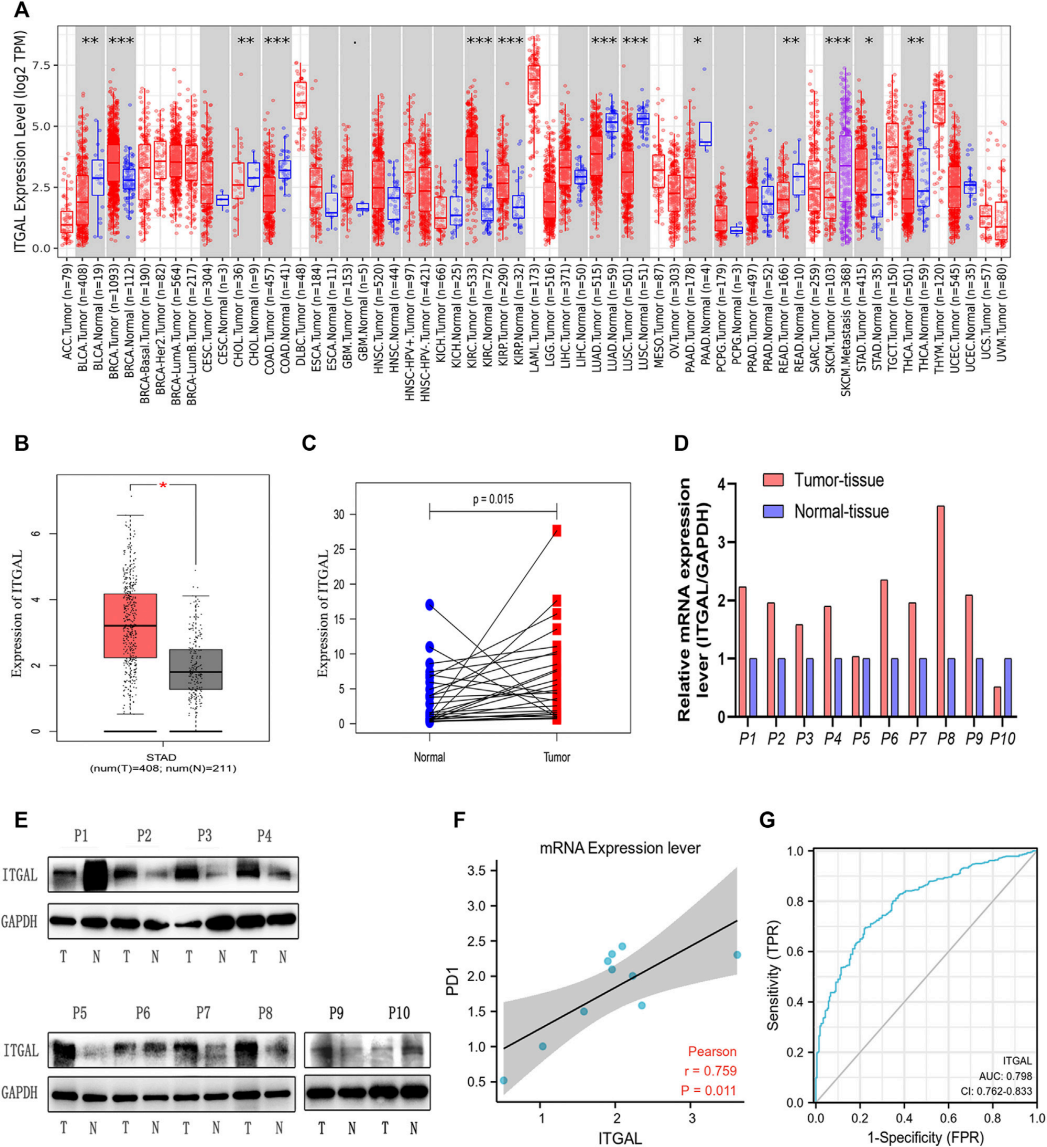
Integrin alpha L (ITGAL) expression levels in different types of human cancers. **(A)** Increased or decreased ITGAL in different tumor types from The Cancer Genome Atlas (TCGA) database were determined by Tumor Immune Estimation Resource (TIMER) (*p < 0.05, **p < 0.01, ***p < 0.001). **(B)** Increased ITGAL in gastric cancer tissues compared with normal tissues in Gene Expression Profiling Interaction Analysis (GEPIA). **(C)** Increased ITGAL expression in gastric cancer compared with the matching normal tissue from TCGA database (*n* = 27). **(D)** The mRNA level of ITGAL in 10 pairs of GC tissues and their paired normal adjacent tissues. **(E)** Western blotting was used to detect the protein level of ITGAL in 10 pairs of GC tissues and their paired adjacent normal tissues. **(F)** The correlation analysis between ITGAL and PD1 mRNA level. **(G)** The receiver-operating characteristic (ROC) curve analysis of ITGAL in gastric cancer (GC) patients.

By applying RNA-seq data from various cancer types in the TCGA, we investigated the differential expression of ITGAL between tumor and surrounding healthy tissues. Figure 1A shows the findings. According to the TIMER database, we discovered that ITGAL expression levels are increased in breast invasive carcinoma, kidney renal clear cell carcinoma, kidney renal papillary cell carcinoma, and stomach adenocarcinoma (STAD), but are decreased in colon adenocarcinoma and lung adenocarcinoma compared with peritumor tissues.

To verify these findings in GC, 619 samples from the TCGA database were examined by GEPIA. As Figure 1B shows, we discovered that ITGAL mRNA was substantially greater in GC samples (408 cases) than that in healthy samples (211cases) (*p* < 0.05) from TCGA, which was similar with the TIMER database. As we can see from Figure 1C, TCGA database showed that ITGAL expression was higher than the matching normal tissue (*n* = 27). Meanwhile, Western blotting and qRT-PCR were performed to examine the ITGAL expression in 10 pairs of GC cases and adjacent healthy tissues. We observed that in most GC tissues, ITGAL mRNA (Figure 1D) and protein levels (Figure 1E) were higher than the paired adjacent healthy tissues. Interestingly, increased ITGAL mRNA expression was consistent with PD1 (Figure 1F), and the AUC was 0.798 (95% CI 0.762–0.833) for ITGAL in GC (Figure 1G). The above data indicated that ITGAL was strongly increased in GC tissues and may be potential diagnostic biomarker for GC.

### Relationship between integrin alpha L expression and gastric cancer patient clinical pathology

**FIGURE 2:**
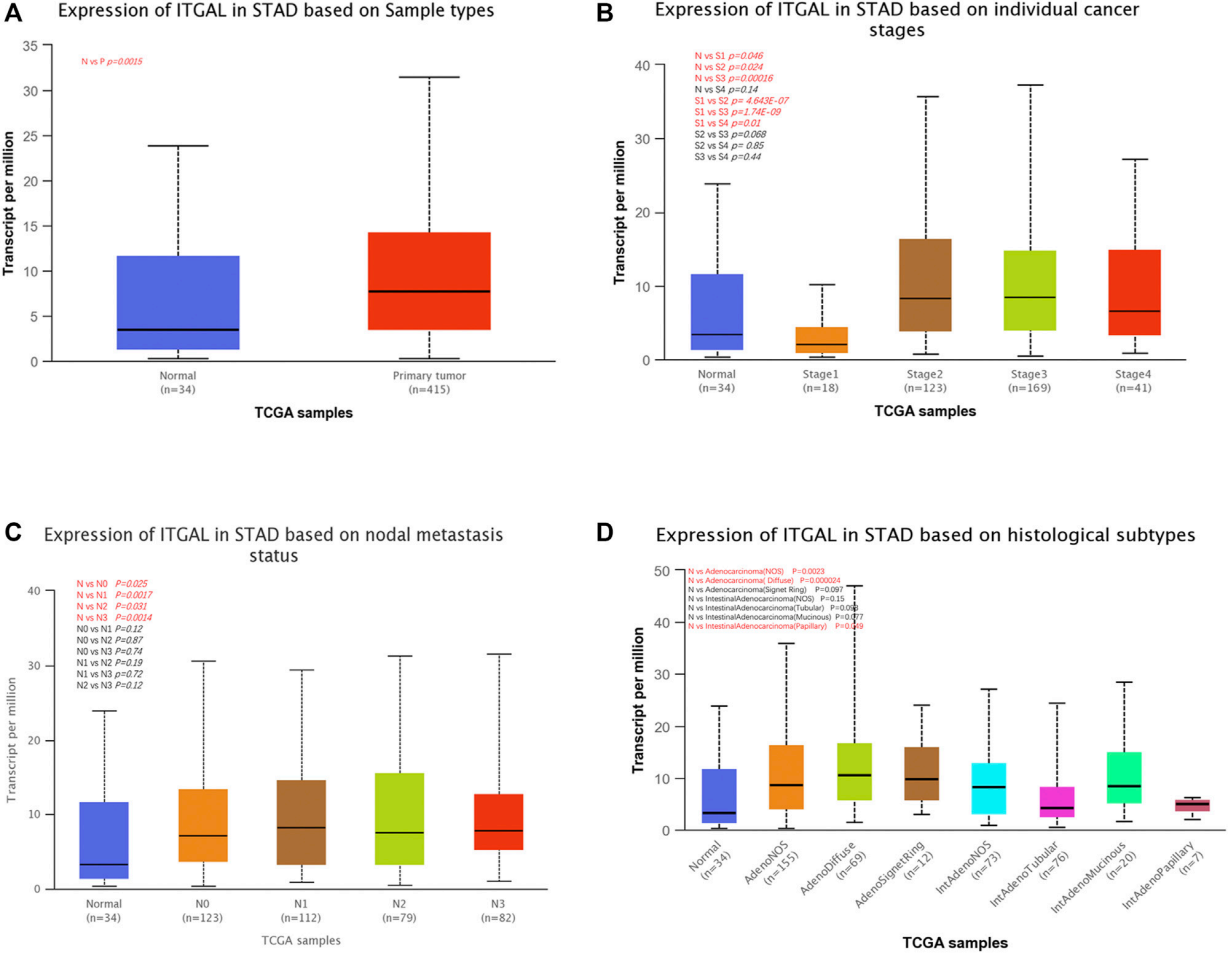
Correlation between ITGAL mRNA expression level and clinicopathological parameters of gastric cancer through the UALCAN database. **(A)** Sample type (normal/primary tumor). **(B)** Cancer stage (stage 1, 2, 3, and 4). **(C)** Lymph node stage (N0 1, 2, and 3). **(D)** Gastric cancer subgroup. N, normal; P, primary tumor; S1, stage 1; S2, stage 2; S3, stage 3; S4, stage 4; STAD, stomach adenocarcinoma.

We examined ITGAL expression in relation to several clinical–pathological parameters in patients, including sample type (healthy/primary tumor), tumor stage (stage 1, 2, 3, and 4), lymph node stage (N0 1, 2, and 3), and gastric cancer subgroup by applying UALCAN. As shown in Figure 2A, GC samples had substantially higher ITGAL expression than that in healthy samples (*p* = 0.0015). A study of cancer stages revealed that ITGAL in the middle and late-stage cancers was significantly higher expressed than in the early stages, suggesting a potential function for ITGAL in tumor development and migration (Figure 2B). ITGAL expression in lymph node stage samples was markedly higher in lymph nodes at all stages of cancer development than normal, indicating that ITGAL is present in malignancy (Figure 2C). Furthermore, as demonstrated in Figure 2D, ITGAL expression was significantly elevated in diffuse adenocarcinoma compared with normal tissue.

Next, in order to get a well grasp of the significance and possible molecular mechanism of ITGAL expression in tumor development, we observed the correlation between the ITGAL expression and clinical–pathological features of GC in the KM plotter. Upregulated ITGAL expression was linked with a poorer OS and PPS in male and female patients. Specifically, increased ITGAL mRNA expression was associated with poorer OS and PPS in stage 1 (OS HR = 0.22, *p* = 0.0062) and stage 2 (OS HR = 2.18, *p* = 0.022; PPS HR = 3.58, *p* = 0.00016) of GC patients (Table 1). Furthermore, we discovered that OS and PPS at stage N1 (OS HR = 2.2, *p* = 0.00082; PPS HR = 3.55, *p* = 2.1e−08) and N1 + 2 + 3 (OS HR = 1.55, *p* = 0.001; PPS HR = 1.94, *p* = 3.9e−06) were related to ITGAL expression at the same time. These findings suggested that the prognostic significance of ITGAL in GC patients was determined by their clinical features, particularly in early-stage and LN metastases of GC.

**TABLE 1:** Correlation of integrin alpha L (ITGAL) mRNA expression and clinical prognosis in gastric cancer with different clinicopathological factors by Kaplan–Meier plotter.

Clinicopathological characteristics	Overall survival (*n* = 881)			Post-progression survival (*n* = 503)		
	N	Hazard ratio	p	N	Hazard ratio	p
**Sex**						
Female	187	1.6 (1.03–2.47)	**0.033**	127	2.63 (1.6–4.31)	**7.1e−05**
Male	349	1.4 (1.03–1.92)	**0.032**	256	1.72 (1.17–2.52)	**0.0055**
**Stage**						
1	62	0.22 (0.07–0.72)	**0.0062**	31	0.54 (0.12–2.43)	0.41
2	135	2.18 (1.1–4.33)	**0.022**	105	3.58 (1.77–7.23)	**0.00016**
3	197	0.7 (0.47–1.05)	0.084	142	1.3 (0.86–1.99)	0.22
4	140	1.44 (0.93–2.23)	0.09	104	2 (1.14–3.54)	**0.015**
**Stage T**						
2	241	1.42 (0.92–2.18)	0.11	196	2.19 (1.39–3.45)	**0.00055**
3	204	1.31 (0.93–1.85)	0.12	150	1.49 (1–2.23)	**0.046**
4	28	1.86 (0.77–4.52)	0.16	29	0.6 (0.24–1.5)	0.27
**Stage N**						
0	74	0.42 (0.17–1.03)	**0.05**	41	1.58 (0.49–5.03)	0.44
1	225	2.2 (1.37–3.53)	**0.00082**	169	3.55 (2.22–5.69)	**2.1e−08**
2	121	0.69 (0.41–1.13)	0.14	105	0.55 (0.32–0.93)	**0.025**
3	76	1.78 (0.92–3.45)	0.085	63	1.69 (0.86–3.33)	0.12
1 + 2 3	422	1.55 (1.19–2.01)	**0.001**	337	1.94 (1.46–2.58)	**3.9e−06**
**Stage M**						
0	444	1.29 (0.98–1.71)	0.067	342	1.85 (1.37–2.5)	**4.2e−05**
1	56	1.36 (0.7–2.62)	0.36	36	2.33 (0.94–5.81)	0.062
**Lauren classification**						
Intestinal	269	1.61 (1.12–2.33)	**0.0098**	192	1.93 (1.26–2.95)	**0.002**
Diffuse	240	1.24 (0.87–1.76)	0.23	176	1.71 (1.16–2.51)	**0.0064**
Mixed	29	0.18 (0.05–0.67)	**0.0042**	16	-	-
**Differentiation**						
** Differentiation**	121	0.53 (0.33–0.87)	**0.01**	49	0.76 (0.36–1.62)	0.48
Moderately	67	1.62 (0.83–3.17)	0.15	24	0.41 (0.15–1.08)	0.062
**HER2**						
Positive	202	1.59 (1.09–2.34)	**0.016**	101	2.43 (1.46–4.07)	**0.00048**
Negative	429	1.41 (1.08–1.85)	**0.012**	283	1.81 (1.3–2.53)	**0.00034**

Note. Bold values indicate *p* < 0.05.

### Increased integrin alpha L mRNA expression linked to poor overall survival in gastric cancer patients

**FIGURE 3:**
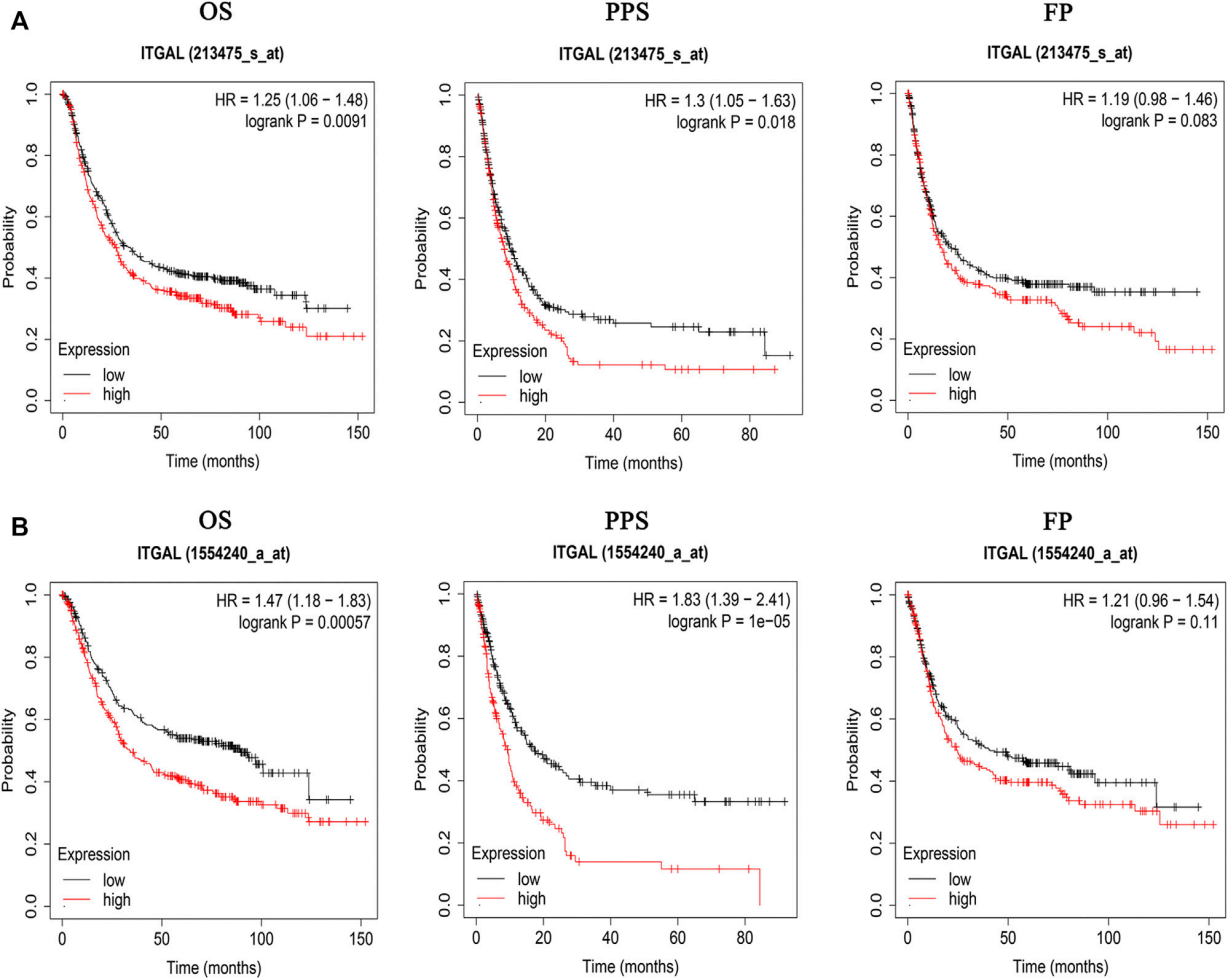
Kaplan–Meier survival curves comparing the high and low expression of ITGAL in gastric cancer in Kaplan–Meier plotter databases. **(A)** Survival curves of OS, PPS, and FP in the gastric cancer cohort (213475-s-at). **(B)** OS, PPS, and FP survival curves of gastric cancer (1554240-a-at). OS, overall survival; PPS, post-progression survival; FP, first progression.

The findings indicated that GC patients had a higher degree of ITGAL mRNA expression compared with normal controls. Therefore, more investigation was necessary to determine whether ITGAL expression is associated with tumor outcome. In the present research, we examined the ITGAL expression and their relationship with prognosis *via* Kaplan–Meier survival curves in order to establish whether ITGAL can be used as a prognostic biomarker in GC. Notably, ITGAL expression was associated with a favorable outcome for GC, and this study showed that increased ITGAL expression was linked with a worse prognosis in the GC cohort 213475-s-at (OS: HR = 1.25, *p* = 0.0091) and 1554240-a-at (OS: HR = 1.47, *p* = 0.00057) (Figure 3).

### Correlation between immune infiltration and integrin alpha L expression in gastric cancer

**FIGURE 4:**
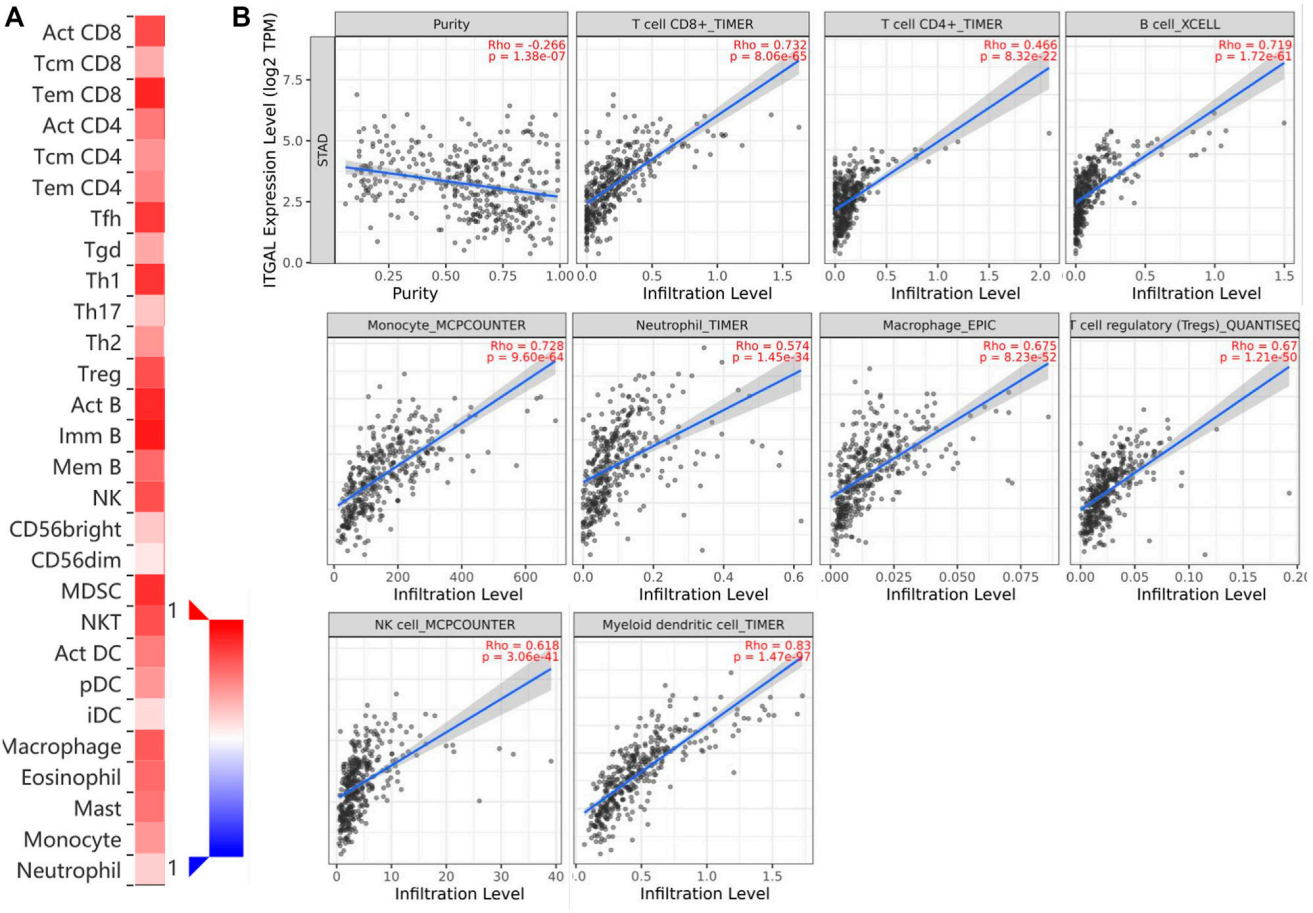
Correlation of ITGAL expression with immune infiltration in gastric cancer. **(A)** Correlation between the expression of ITGAL and the abundance of TILs in gastric cancer available at TISIDB database. **(B)** Correlation of ITGAL expression with infiltration levels of CD8 + T cell, CD4 + T cell, Treg cell, B cell, neutrophil, macrophage, myeloid dendritic cell, natural killer cell, and monocyte in gastric cancer available at TIMER2.0 database. TILs, tumor-infiltrating lymphocytes; TIMER2.0, Tumor Immune Estimation Resource. Color images are available online.

Immune infiltration is a key factor associated with tumor progression. Therefore, TISIDB and TIMER platforms were performed to assess ITGAL expression connection to immune cell infiltration levels in STAD. The purity of tumors in clinical cancer samples has a significant impact on the study of immune infiltration using genetic techniques (Yoshihara et al., 2013). Therefore, in this study, ITGAL expression is adversely correlated with the purity of STAD (rho = -0.266, *p* <1.38e−7). Our results also discovered that ITGAL had a strong correlation with the abundance of TILs (Figure 4A). For instance, high-expression level of ITGAL was positively correlated with infiltrating degree of CD8^+^ T cell (rho = 0.732), CD4^+^ T cell (rho = 0.466), B cell (rho = 0.719), monocyte (rho = 0.728), neutrophil (rho = 0.574), and macrophage (rho = 0.675), T-cell regulatory (rho = 0.67), NK cell (rho = 0.618), and myeloid dendritic cell (rho = 0.8) (Figure 4B). All the *p*-values were far less than 0.001. These results indicate that ITGAL plays a key function in immune infiltration of GC.

TIMER and GEPIA databases were applied to study the association among ITGAL and different biomarkers of TILs (CD8+/CD4+ T cells, NK cells, B cells, monocytes, DCs, TAMs, M1macrophages, M2 macrophages, neutrophils, T cells, and related subtypes) in STAD. We found that ITGAL was associated with the majority of TILs markers in STAD. The several functional T cells, including Th1/Th2/Th17/Tfh cells, Tregs, and exhausted T cells were also analyzed. Particularly, ITGAL was strongly linked with the majority of immune marker sets of TILs in STAD (Table 2).

**TABLE 2:** Correlation analysis between ITGAL and related genes and markers of immune cells in Tumor Immune Estimation Resource (TIMER2.0).

Description	Gene markers	STAD			
		None		Purity	
		Cor	*p*	Cor	*p*
CD8^+^ T cell	CD8A	0.845	***	0.83	***
	CD8B	0.658	***	0.64	***
T cell (general)	CD3D	0.873	***	0.856	***
	CD3E	0.904	***	0.894	***
	CD2	0.691	***	0.88	***
B cell	CD19	0.893	***	-	-
	CD79A	0.711	***	0.683	***
Monocyte	CD86	0.75	***	0.733	***
	CD115 (CSF1R)	0.712	***	0.716	***
TAM	CCL2	0.414	***	0.373	***
	CD68	0.498	***	0.49	***
	IL10	0.556	***	0.53	***
M1 Macrophage	INOS (NOS2)	0.146	*	0.139	*
	IRF5	0.42	***	0.399	***
	COX2(PTGS2)	−0.053	0.28	−0.098	0.0561
M2 Macrophage	CD163	0.631	***	0.626	***
	VSIG4	0.549	***	0.546	***
	MS4A4A	0.669	***	0.657	***
Neutrophils	CD66b	−0.001	0.978	−0.009	0.859
	CD11b (ITGAM)	0.666	***	0.656	***
	CCR7	0.784	***	0.76	***
Natural killer cell	KIR2DL1	0.339	***	0.312	***
	KIR2DL3	0.31	***	0.269	***
	KIR2DL4	0.416	***	0.382	***
	KIR3DL1	0.39	***	0.382	***
	KIR3DL2	0.481	***	0.45	***
	KIR3DL3	0.137	*	0.153	*
	KIR2DS4	0.341	***	0.321	***
Dendritic cell	HLA-DPB1	0.791	***	0.771	***
	HLA-DQB1	0.616	***	0.57	***
	HLA-DRA	0.733	***	0.71	***
	HLA-DPA1	0.722	***	0.696	***
	BDCA-1(CD1C)	0.616	***	0.583	***
	BDCA-4(NRP1)	0.469	***	0.447	***
	CD11c (ITGAX)	0.718	***	0.713	***
Th1	T -bet (TBX21)	0.882	***	0.877	***
	STAT4	0.83	***	0.826	***
	STAT1	0.527	***	0.533	***
	IFN-γ (IFNG)	0.605	***	0.59	***
	TNF-α (TNF)	0.356	***	0.286	***
Th2	GATA3	0.64	***	0.616	***
	STAT6	0.287	***	0.313	***
	STAT5A	0.631	***	0.622	***
	IL13	0.183	**	0.173	**
Tfh	BCL6	0.324	***	0.301	***
	IL21	0.531	***	0.511	***
Th17	STAT3	0.411	***	0.416	***
	IL17A	0.1	0.0417	0.095	0.0651
Treg	FOXP3	0.801	***	0.779	***
	CCR8	0.738	***	0.733	***
	STAT5B	0.485	***	0.509	***
	TGFβ (TGFB1)	0.499	***	0.485	***
T cell exhaustion	PD-1 (PDCD1)	0.799	***	0.78	***
	PDL1(PDCD1LG2)	0.757	***	0.745	***
	CTLA4	0.702	***	0.668	***
	LAG3	0.735	***	0.718	***
	TIM-3 (HAVCR2)	0.751	***	0.74	***
	GZMB	0.562	***	0.523	***

Note. STAD, stomach adenocarcinoma; TAM, tumor-associated macrophage; Th, T helper cell; Tfh, Follicular helper T cell; Treg, regulatory T cell; Cor, R value of Spearman’s correlation; None, correlation without adjustment; Purity; correlation adjusted by purity.

*p < 0.01; **p < 0.001; ***p < 0.0001.

**FIGURE 5:**
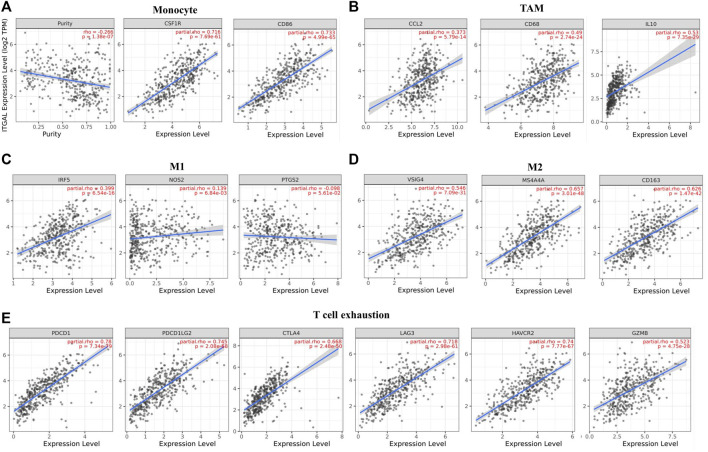
ITGAL expression correlated with monocyte, macrophages, and T cell exhaustion in stomach adenocarcinoma (STAD). Markers include CD86 and CSF1R of monocytes; CCL2, CD68, and IL10 of TAMs; NOS2, IRF5, and PTGS2 of M1 macrophages; and CD163, VSIG4, and MS4A4A of M2 macrophages. Scatterplots of correlations between ITGAL expression and gene markers of monocytes **(A)**, TAMs **(B)**, and M1 **(C)**, M2 macrophages **(D)** and T-cell exhaustion **(E)** in STAD (*n* = 415).

Obviously, ITGAL had a significant association with the majority of marker sets of monocytes, TAMs, M2 macrophages, and T cell exhaustion in STAD (Table 2). Specifically, this study implicated that PDCD1, PDCD1G2, CTLA4, LAG3, HAVCR2, GZMB of T-cell exhaustion, and chemokine ligand (CCL)-2, CD68, and IL10 of TAMs are all strongly correlated with ITGAL in STAD, as well as IRF5 of M1 phenotype and CD163, VSIG4, MS4A4A of M2 phenotype (*p* < 0.0001; Figures 5A–E). Furthermore, according to the GEPIA database, we further assessed the connection of ITGAL expression with the aforementioned markers of TAMs, M2 macrophages, monocytes, and T-cell exhaustion, and the correlations are comparable with that in TIMER (Table 3). Therefore, ITGAL may regulate T-cell exhaustion, and macrophage polarization in STAD.

**TABLE 3:** Correlation analysis between ITGAL and related genes and markers of monocyte, macrophages, and T-cell exhaustion in Gene Expression Profiling Interaction Analysis (GEPIA).

Description	Gene markers	STAD			
		Tumor		Normal	
		Cor	*p*	Cor	*p*
Monocyte	CD86	0.75	***	0.79	***
	CD115 (CSF1R)	0.72	***	0.68	***
TAM	CCL2	0.41	***	−0.13	0.45
	CD68	0.47	***	0.45	*
	IL10	0.58	***	0.54	**
M1 Macrophage	INOS (NOS2)	0.16	**	0.16	0.36
	IRF5	0.4	***	0.32	0.06
	COX2(PTGS2)	0.035	0.48	−0.41	0.43
M2 Macrophage	CD163	0.59	***	−0.082	0.64
	VSIG4	0.54	***	0.22	0.2
	MS4A4A	0.68	***	0.14	0.4
T cell exhaustion	PD-1 (PDCD1)	0.75	***	0.88	***
	PDL1(PDCD1LG2)	0.78	***	0.44	*
	CTLA4	0.7	***	0.84	***
	LAG3	0.66	***	0.79	***
	TIM-3 (HAVCR2)	0.76	***	0.74	***
	GZMB	0.5	***	0.71	***

Note. STAD, stomach adenocarcinoma; TAM, tumor-associated macrophages; Tumor, correlation analysis in tumor tissue of TCGA; Normal, correlation analysis in normal tissue of TCGA.

*p < 0.01; **p < 0.001; ***p < 0.0001.

### The expression of integrin alpha L is associated with immunomodulators in gastric cancer

**FIGURE 6:**
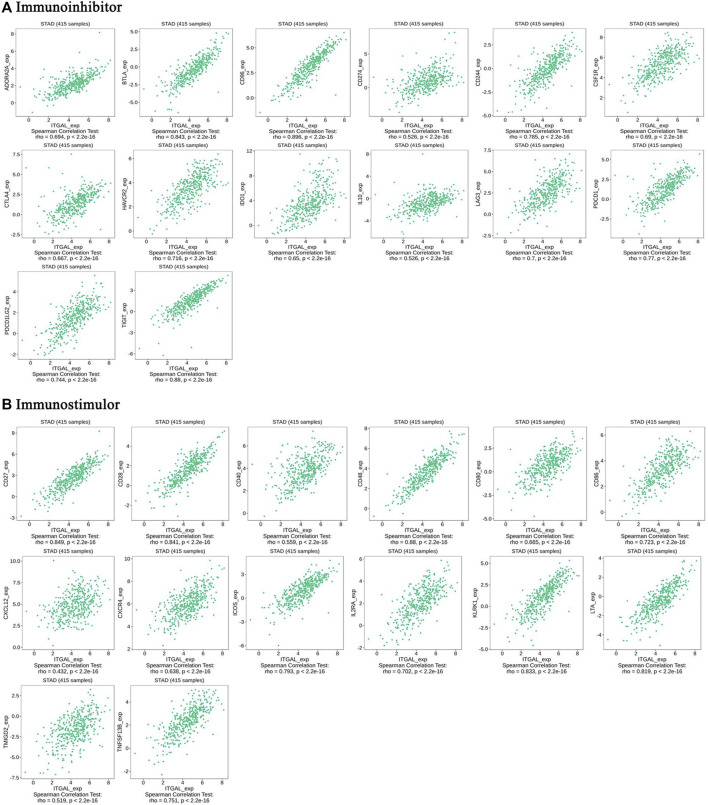
The expression of ITGAL is associated with immunomodulators in gastric cancer. **(A)** Correlation between ITGAL expression and immunoinhibitors in gastric cancer available at TISIDB database. **(B)** Correlation between ITGAL expression and immunostimulators in gastric cancer available at TISIDB database. Color images are available online.

Immunomodulators are substances that affect the function of the immune system. This research indicated that ITGAL was significantly connected with immunoinhibitors (*p* < 2.2e−16), such as BTLA (rho = 0.843), CD96 (rho = 0.89), CD274 (rho = 0.526), CSF1R (rho = 0.69), HAVCR2 (rho = 0.716), PDCD1 (rho = 0.77), and TIGIT (rho = 0.88) (Figure 6A). The expression of ITGAL was also closely associated with immunostimulators (*p* < 2.2e−16), including CD27 (rho = 0.849), CD28 (rho = 0.841), CD40 (rho = 0.559), CD48 (rho = 0.88), CD80 (rho = 0.665), CD86 (rho = 0.723), CXCR4 (rho = 0.638), ICOS (rho = 0.793), IL2RA (rho = 0.702), KLRK1 (rho = 0.833), and LTA (rho = 0.819) (Figure 6B). These results suggested that ITGAL is intimately engaged in the regulation of the immune interaction and may modulate tumor immune escape.

### Correlation between the integrin alpha L expression and chemokines in gastric cancer

**FIGURE 7:**
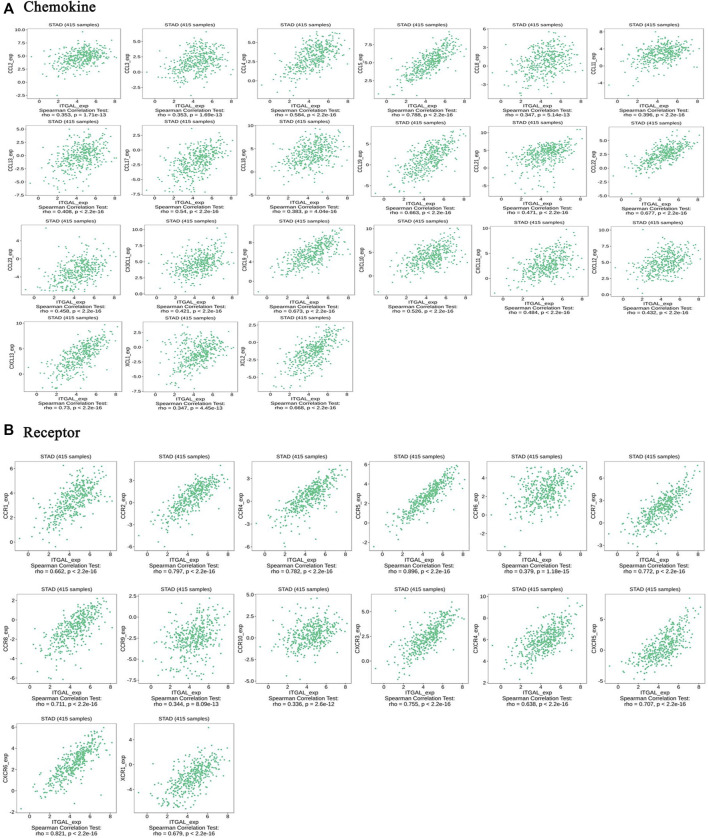
Correlation between the expression of ITGAL and chemokines in gastric cancer. **(A)** Correlation between ITGAL expression and chemokines in gastric cancer available at TISIDB database. **(B)** Correlation between ITGAL expression and chemokine receptors in gastric cancer available at TISIDB database. Color images are available online.

Chemokines play a great function in controlling infiltration degree of immune cell. This research implicated the association between ITGAL expression with chemokines. For example, ITGAL expression was significantly linked with CCL2 (rho = 0.353), CCL3 (rho = 0.353), CCL4 (rho = 0.584), CCL5 (rho = 0.788), CCL8 (rho = 0.347), CCL11 (rho = 0.396), CCL13 (rho = 0.408), CCL17 (rho = 0.54), CCL18 (rho = 0.383), CCL19 (rho = 0.663), CCL21 (rho = 0.471), CCL22 (rho = 0.677), CCL23 (rho = 0.458), CX3CL1 (rho = 0.421), CXCL9 (rho = 0.673), CXCL10 (rho = 0.526), CXCL13 (rho = 0.73), and XCL2 (rho = 0.668) (Figure 7A). All the values of p were far less than 0.001. Meanwhile, we demonstrated that ITGAL expression was also significantly correlated with chemokine receptors (*p* < 0.001), including CCR1 (rho = 0.662), CCR2 (rho = 0.797), CCR4 (rho = 0.782), CCR5 (rho = 0.896), CCR6 (rho = 0.379), CCR7 (rho = 0.772), CCR8 (rho = 0.711), CCR9 (rho = 0.344), CCR10 (rho = 0.336), CXCR3 (rho = 0.755), CXCR4 (rho = 0.638), CXCR5 (rho = 0.707), CXCR6 (rho = 0.821), and XCR1 (rho = 0.679) (Figure 7B). These results further demonstrated the findings that ITGAL may function as an immunoregulatory factor in GC.

### Correlation between integrin alpha L expression and infiltration degree of T-cell exhaustion in gastric cancer

**FIGURE 8:**
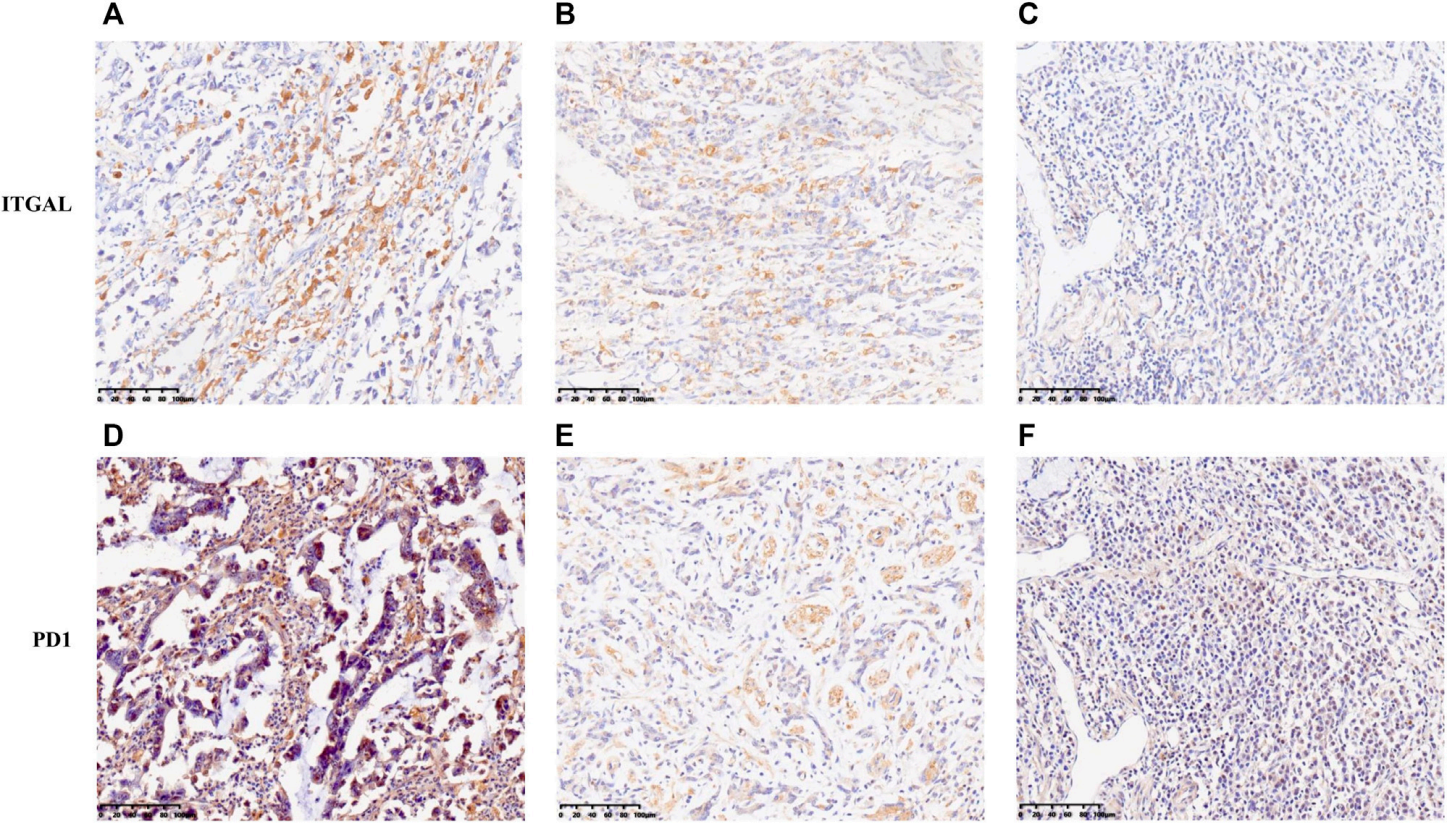
Correlation between ITGAL expression and immune infiltration levers of T-cell exhaustion in gastric cancer. The expression of ITGAL [**(A)** +++, **(B)** ++, **(C)** +]. The expression of PD1 [**(D)** +++, **(E)** ++, **(F)** +]. The expression density of ITGAL and PD1 in gastric cancer tissue was quantitated by scoring staining intensity, including negative (−) and weak (+) staining, moderate (++) and strong (+ + +) staining, respectively.

In this research, TIMER and GEPIA databases were performed to investigate whether ITGAL expression had a correlation with T-cell exhaustion infiltration in GC (Figure 5 **and** Table 3), and ITGAL expression was positively correlated with PD1 (PDCD1) in GC. Therefore, we conducted an immunohistochemical method to determine the association between ITGAL and PD1 expression, and the results are presented in Figure 8. By scoring staining intensity, we classified the levels of the expression into four groups: negative (−), weak (+), moderate (++), and strong (+ + +) staining. ITGAL showed strong expression in most GC specimens, and a few specimens showed weak staining. Obviously, PD1 was consistent with ITGAL staining intensity (Supplementary Figure S1). The expression level of PD1 was correspondingly high and low in ITGAL strong and weak expression samples. These findings further demonstrated that the upregulated ITGAL corresponds to a higher T-cell exhaustion infiltration degree.

## Discussion

In the present research, a comprehensive bioinformatics investigation was performed to systematically analyze the clinical significance and expression lever of ITGAL in GC. Our analyses revealed poor prognosis was consistent with high expression of ITGAL in GC. Furthermore, our data also indicated that ITGAL expression had a close association with infiltration degrees of different immune cells, immunostimulators, immunoinhibitors, chemokines, and receptors in GC. Therefore, our research revealed new insights in understanding the critical function of ITGAL, and it may be a prognostic biomarker linked with immune infiltration of GC.

Integrins, the heterodimers produced by α and β subunit noncovalent binding, not only can regulate intercellular function including cellular adhesion, cell–matrix adhesion, and tumor microenvironment but also control cell proliferation and migration by recognizing specific extracellular ligands (Ruoslahti and Pierschbacher, 1987; Anikeeva et al., 2005; Schwartz et al., 2018). ITGAL, as a member of the integrin family, plays a key function in a variety of immunological processes, including leukocyte–endothelial cell interaction and cytotoxic T-cell-mediated killing (Barber et al., 2004; Giblin and Lemieux, 2006). Although ITGAL has not been thoroughly investigated, it is known that the carcinogenic potential of ITGAL, whose expression levels of ITGAL plays a crucial role in carcinogenic potential, was correlated with renal cancer, ovarian cancer, colorectal cancer, and head and neck squamous cell carcinoma (Vendrell et al., 2007; Boguslawska et al., 2016; Song et al., 2019; Zhao et al., 2019; Ji et al., 2020). However, the possible function of ITGAL in regulating tumor immunity and its clinical significance in GC are still unknown.

Thus, we evaluated ITGAL expression of GC by dependent databases including GEPIA, TIMER, TCGA, and UALCAN. We discovered that ITGAL was an aberrant expression between cancer and paracancerous tissues in various malignancies. Moreover, ITGAL was obviously increased in GC compared with paracancerous samples. These results were consistent with those from TCGA database. We also found that the ITGAL mRNA and protein levels were increased in most GC samples compared with the paired paracancerous samples. These results show that the level of ITGAL expression may serve as a potential diagnostic indicator in GC. Furthermore, to confirm whether ITGAL can be used as a prognostic biomarker, we used the KM plotter database to analyze the correlation between the ITGAL expression and OS, PPS, and FP in GC cohorts (213475-s-at and 1554240-a-at). Notably, analysis of this database indicated that the higher ITGAL expression correlated with HR for worse OS and poor PPS of GC. In addition, upregulated ITGAL expression had a significant correlation with a worse prognosis of GC in stages 1 and 2, N1 and N1 + 2 + 3 with the highest HR for worse OS and in stages 2 and 4, T2 to T3, N1, N2, and N1 + 2 + 3 for worse PPS. Together, these observations strongly support our hypothesis that ITGAL is a prognostic biomarker in GC.

Additionally, this study discovered that ITGAL is strongly related to the degree of immune infiltration in GC. In the cancer microenvironment, it has been demonstrated that immune cell infiltration plays critical roles in the development and progression of cancers (Curigliano, 2018; Klauschen et al., 2018). ITGAL is a tissue-specific integrin that plays a role in inflammatory and immune responses (Lu et al., 2002). Recent studies have revealed that ITGAL could promote T-cell migration *via* reaction to Rho GTPase signaling suppression caused direct CLL cell contact (Ramsay et al., 2013; Haspels et al., 2018). However, whether ITGAL expression is linked with immune infiltration in GC remains unknown. Therefore, we systematically examined the association between ITGAL expression and the degree of immune infiltration in GC. In our experience, this is the first time that ITGAL regulating immune infiltration with GC is identified. Our study showed that ITGAL expression had a strong correlation with TILs including CD8+T cell, CD4+T cell, Treg cell, B cell, neutrophil, TAM, DCs, NK cell, and monocyte. At the same time, increased ITGAL expression was associated with immunostimulators, immunoinhibitors, chemokines, and receptors. In addition, this study also demonstrated the association between ITGAL expression and the TIL marker genes of GC. Obviously, ITGAL expression had an association with M2 macrophage markers, including CD163, VSIG4, and MS4A4A, whereas M1 macrophage markers, such as NOS2 and IRF5, correlated with ITGAL expression in very weak and moderate ways correspondingly. Macrophages, a kind of specialized immune cells, are divided into M1 and M2 macrophages (Jayasingam et al., 2019), and play a crucial function in proliferation (Su et al., 2017), angiogenesis (Sammarco et al., 2018), invasion (Zhang et al., 2018), and metastasis (Song et al., 2017), and immunity of tumor (Wynn et al., 2013). These findings indicate that ITGAL has a potential function to regulate the polarization of TAMs.

Furthermore, upregulated ITGAL expression was strongly correlated with Tregs markers (FOXP3, CCR8) and T-cell exhaustion markers (PD1, CTLA4, LAG3). Immune checkpoint blockade is the main immunotherapeutic strategy. PD1/PDL1, as the important immune checkpoint component, has been verified to regulate the function of TILs. To date, PD1/PDL1 checkpoint blockade therapy is widely performed to various malignancies including GC (Kang et al., 2017; Fuchs et al., 2018; Kim et al., 2018), but some studies discovered that PD-1 has a critical function in tumor antigen tolerance, leading to poor therapeutic effect in some patients with PD1 therapy (Woo et al., 2012; Kang et al., 2017). Therefore, the most important is to improve tumor cell response to immune checkpoint inhibitors and cytokines. Our results revealed that increased ITGAL expression was not only associated with PD1 and CTLA4 but also significantly correlated with cell response to chemokines according to the TISIDB, TIMER, and GEPIA databases. These results reflected that it may be a strategy for enhancing immunotherapy effectiveness by targeting ITGAL. In conjunction with these results, ITGAL played a vital function in recruiting and modulating TILs in GC, and it is worth to continue investigating the molecular mechanism and function of ITGAL in modulating tumor microenvironment.

However, our study has some limitations. A limitation of this study is that most data are based on the online platform databases, which are updated and expanded continuously; therefore, the results of the research may be affected. Second, the information of complications and treatment option is not included in our study. Third, the *in vitro* and *in vivo* experiment is not used to validate the function of ITGAL in GC and the molecular mechanism of ITGAL in GC immunity, but in the future study, we guarantee that we will put more emphasis to the whole baseline information of patients, and experiments will be performed to further validate the projected results.

## Conclusion

The upregulated ITGAL expression is closely correlated with poor prognosis and enhanced immune infiltration degree including CD8+ T cells, C4+ T cells, macrophages, neutrophils, and myeloid dendritic cells in GC. Moreover, the expression of ITGAL contributes to the regulation of M2 macrophages, Treg, and T-cell exhaustion. Therefore, this study suggests that ITGAL may, as a prognosis biomarker, highlight its novel potential function in the regulation of immune cell infiltration in GC patients.

## Data Availability

The original contributions presented in the study are included in the article/Supplementary Material, further inquiries can be directed to the corresponding authors.

## Supplementary Material

Supplementary Figure 1 Correlation between ITGAL and PD1 expression based on immunohistochemical score.
