# Supplementary figures and images for: ITGAL as a Prognostic Biomarker Correlated With Immune Infiltrates in Gastric Cancer
Source: Front Cell Dev Biol. 2022 Mar 24;10:808212. doi: 10.3389/fcell.2022.808212 (PMC8987306; doi:10.3389/fcell.2022.808212)

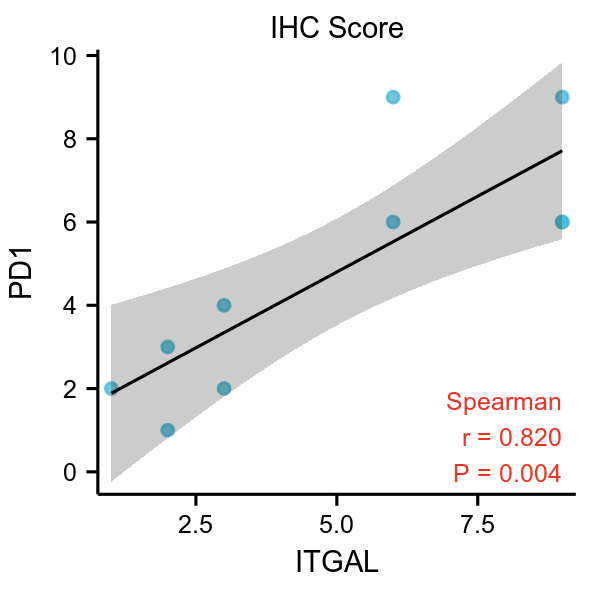

Supplement: Supplementary file 1 [file Image1.TIFF]
